# Comprehensive Cross-Sectional Evaluation of Human Sandfly-Borne Phlebovirus Exposure in an Endemic Region

**DOI:** 10.3390/v15091902

**Published:** 2023-09-09

**Authors:** Ceylan Polat, Nazlı Ayhan, Mehmet Bakır Saygan, Sevilay Karahan, Remi Charrel, Koray Ergünay

**Affiliations:** 1 Department of Medical Microbiology, Faculty of Medicine, Hacettepe University, Ankara 06230, Turkey; ergunayk@si.edu; 2 Unité des Virus Emergents, Aix Marseille University, IRD 190, INSERM U1207, 13005 Marseille, France; nazliayhann@gmail.com (N.A.); remi.charrel@univ-amu.fr (R.C.); 3 National Reference Center for Arboviruses, National Institute of Health and Medical Research (Inserm) and French Armed Forces Biomedical Research Institute (IRBA), 13005 Marseille, France; 4 Middle Anatolia Regional Blood Center, Turkish Red Crescent Society, Ankara 06378, Turkey; mehmet.saygan@kizilay.org.tr; 5 Department of Biostatistics, Faculty of Medicine, Hacettepe University, Ankara 06230, Turkey; skarahan@hacettepe.edu.tr; 6 Laboratoire des Infections Virales Aigues et Tropicales, Pole des Maladies Infectieuses, AP-HM Hopitaux Universitaires de Marseille, 13005 Marseille, France; 7 Walter Reed Biosystematics Unit (WRBU), Smithsonian Institution Museum Support Center, Suitland, MD 20746, USA; 8 One Health Branch, Walter Reed Army Institute of Research (WRAIR), Silver Spring, MD 20910, USA; 9 Department of Entomology, Smithsonian Institution-National Museum of Natural History (NMNH), Washington, DC 20560, USA

**Keywords:** phlebovirus, sandfly fever, Toscana virus, microneutralization, serology

## Abstract

Sandfly-borne phleboviruses are endemic in countries around the Mediterranean Basin and pose a significant health threat for populations, with symptoms spanning from febrile diseases to central nervous system involvement. We carried out a comprehensive cross-sectional screening via microneutralization (MN) assays for a quantitative assessment of neutralizing antibodies (NAs) to seven phleboviruses representing three distinct serocomplexes, using samples previously screened via immunofluorescence assays (IFAs) in Turkey, an endemic region with various phleboviruses in circulation. We detected NAs to three phleboviruses: Toscana virus (TOSV), sandfly fever Naples virus (SFNV), and sandfly fever Sicilian virus (SFSV), while assays utilizing Adana virus, Punique virus, Massilia virus, and Zerdali virus remained negative. The most frequently observed virus exposure was due to TOSV, with a total prevalence of 22.6%, followed by SFNV (15.3%) and SFSV (12.1%). For each virus, IFA reactivity was significantly associated with NA detection, and further correlated with NA titers. TOSV and SFSV seroreactivities were co-detected, suggesting exposure to multiple pathogenic viruses presumably due to shared sandfly vectors. In 9.6% of the samples, multiple virus exposure was documented. In conclusion, our findings demonstrate widespread exposure to distinct pathogenic phleboviruses, for which diagnostic testing and serological screening efforts should be directed.

## 1. Introduction

Phlebotomine sandflies (Diptera: *Psychodidae*, *Phlebotominae*) are capable of transmitting several microbial pathogens including particular bacteria (e.g., Bartonella), parasites (e.g., Leishmania), and viruses [1]. Viral pathogens vectored by sandflies mainly comprise phleboviruses, currently classified as a separate genus within the *Phenuiviridae* family (order *Bunyavirales*) [2]. As vector arthropods responsible for maintenance and transmission, sandflies have a global distribution spanning southern Europe, Asia, Africa, Australia, and Central and South America, with peak activity in the warmer months of the year [3]. Despite having a relatively limited geographic dispersal capacity compared with mosquitoes, sandflies exhibit an expansion of activity zones owing to changes in the climate and ecosystem, as exemplified by vector abundance and leishmaniasis cases [3,4]. Therefore, increased incidence of infections or introduction of pathogens into naïve regions, leading to disease emergence and outbreaks, should be expected [1,3].

Phleboviruses possess a tripartite RNA genome, comprising L, M, and S segments encoding for the virus replicase (RNA polymerase), glycoprotein precursors, and nucleoprotein, respectively [2]. To date, over 10 phleboviruses have been documented as human pathogens in both hemispheres, and they are likely to increase due to the increased use of diagnostic approaches and natural reassortment events [5]. The majority of phlebovirus infections in exposed individuals result in seroconversion without any clinical disease, which is commonly observed in regions with endemic virus circulation. Symptomatic phlebovirus infections frequently present as a self-limited febrile illness (known as sandfly or three-day fever), typically occurring with a sudden onset of fever accompanied by malaise, anorexia, photophobia, gastrointestinal symptoms, or skin rash [5,6,7]. In the Mediterranean Basin, causative phleboviruses associated with sandfly fever are sandfly fever Sicilian virus (SFSV, *Sicilian phlebovirus* species), sandfly fever Naples virus (SFNV, *Naples phlebovirus* species), and their variants [1,6,7]. Another Old World phlebovirus, Toscana virus (TOSV, *Toscana phlebovirus* species), is unique as an agent of central nervous system infections that manifest as aseptic meningitis, encephalitis, and meningoencephalitis following the febrile period, resulting in better chances for virus-specific diagnosis in a clinical setting in regions with virus circulation [5].

Diagnosis of phlebovirus infections and exposure relies on viremia dynamics and host immune response, where viral (antigens or nucleic acids) and immune response elements (IgM and IgG antibodies) can be utilized as targets for detection [5,6,7]. In general, virus nucleic acids and antigens coincide with viremia and are mostly detectable during the first few days of symptomatic infections. Antibody detection in the blood provides diagnostic utility for a broader timeframe, whereby IgM becomes reactive early within the first week after disease onset and continues to be positive for weeks or months thereafter, while IgG appears and increases within the first several weeks to remain detectable for years following infection, providing an outstanding marker of exposure for serological screening [5,6,7]. For TOSV, IgG avidity was observed to reach a plateau phase at 1–3 months after acute infection and drop substantially within 2 years [8]. Despite high levels of total anti-TOSV IgG, moderate levels of neutralizing antibodies (NAs) have been detected over time, suggesting mechanisms other than direct virus neutralization such as antibody-dependent cellular cytotoxicity and complement-dependent cytotoxicity are involved in re-exposure protection [8].

Various methods can be used for the detection of phlebovirus antibodies, including the hemagglutination inhibition assay, immunofluorescence assay (IFA), enzyme-linked immunosorbent assay (ELISA), immunoblot (IB) assay, and neutralization-based assays [7,9,10]. Some of these assays are commercially available and have been developed utilizing partially purified antigens, infected cells, or recombinant viral proteins. Important setbacks in antibody detection are serological cross-reactions between antigenically related phleboviruses, which typically occur among members of the identical or related virus species—also described as serocomplexes [10,11]. Briefly, pre-existing antibodies against a particular phlebovirus might produce false-positive assay results due to cross-reactivity, despite the lack of any cross-protection between viruses. This is particularly evident when partially purified virus extracts or infected cells are employed as the antigen source for serological testing [9,10]. Neutralization-based assays are generally regarded as the gold standard for the confirmation of phlebovirus antibody specificity [10,11]. Based on immune responses against several antigenic epitopes on viral glycoproteins, neutralization assays remain essential to resolve the complex seroreactivity profiles that are likely to occur due to temporal exposure to several distinct phleboviruses in endemic regions.

Turkey—a Mediterranean country on the Anatolian peninsula and Eastern Thrace—is situated between diverse biogeographical zones and serves as a natural hub for the dispersion of vector-borne viruses among African, Asian, and European continents [12]. Several sandfly-borne viruses have been documented in Anatolia and Eastern Thrace, including TOSV and SFSV clades, as well as novel viruses with unknown public health impact [12]. Initial TOSV cases were reported in 2011, and symptomatic infections were identified in individuals with immunosuppression and in young adults co-infected with West Nile virus [13,14,15]. TOSV was further suggested as a potential precipitating vector-borne virus for Guillain–Barré syndrome in a case–control study [16]. Moreover, symptomatic infections with SFSV and the local SFSV clade—sandfly fever Turkey virus (SFTV)—have been reported, sometimes with severe presentation including gastrointestinal symptoms, postinfectious asthenia syndrome, and even central nervous system involvement [17,18]. Despite several reports of acute infections and preliminary findings of seroreactivity in human and nonhuman vertebrates, information on phlebovirus exposure remains limited from Turkey [12]. In this study, we carried out an extensive microneutralization-based quantitative investigation of exposure in IFA-reactive samples, utilizing several well-known and novel phlebovirus isolates.

## 2. Materials and Methods

### 2.1. Samples

A total of 124 sera were overviewed, collected between January and April 2009 at four major branches of the Turkish Red Crescent Middle Anatolia Regional Blood Center (in Ankara, Konya, Eskisehir and Zonguldak provinces of Turkey), after informed consent was obtained from volunteer blood donors. Sera were included in the study according to IFA findings and availability. The participants underwent a brief medical examination and were afebrile at the time of the sample collection. Mean participant age was 36.0 (range: 19–63; SD: 11.02), with 3.2% being women. All participants filled out a questionnaire to reveal the presence of risk factors for vector-borne infections. The samples were transported on dry ice and stored in aliquots at −80 °C.

### 2.2. Immunofluorescence Assay (IFA)

Sera were tested using a commercial indirect immunofluorescence assay (IFA) for sandfly fever phleboviruses (Sandfly fever virus IgG Mosaic I; EuroImmun, Luebeck, Germany), according to the manufacturer’s recommendations. The assay allows IgG antibody detection for phleboviruses commonly associated with sandfly fever (SFSV, SFNV, and sandfly fever Cyprus virus, SFCV, a variant of SFSV), as well as TOSV, and utilizes cell-culture-grown isolates as the antigen source, located separately on the chip. The results were interpreted via fluorescence microscopy. Antibody levels were determined at the single serum dilution of 1:100. Positive results were interpreted as weak (+), moderate (++), strong (+++), and very strong (++++) according to visual intensity of fluorescence compared with the serially diluted control sera.

### 2.3. Microneutralization Assay (MN)

Each serum was tested for neutralizing antibodies using virus isolates representing three distinct phlebovirus serocomplexes including: (i) TOSV, SFNV, and Zerdali virus (ZERV) (sandfly fever Naples serocomplex); (ii) SFSV (sandfly fever Sicilian serocomplex); and (iii) Adana virus (ADAV) (Salehabad serocomplex). The microneutralization assay (MN) was performed as described previously (10). Briefly, two-fold serial dilutions from 1:20 to 1:160 were prepared for each sample and a volume of 50 μL was pipetted into 96-well plates. Viruses were titrated in Vero cells (ATCC CCL81) and 50 μL of 100 TCID_50_ virus was added into each well except for the controls. The control wells included 50 μL of minimum essential medium (MEM) enriched with 5% fetal bovine serum, 1% penicilin + streptomycin, 1% L-glutamine 200 mM, 1% kanamycin, and 3% fungizone. Following incubation at 37 °C for one hour, a 100 μL suspension of 2 × 10^5^ Vero cells/ mL was added to each well, and the plates were further incubated at 37 °C in the presence of 5% CO_2_. The microplates were visually interpreted under an inverted microscope after five days (for TOSV, ZERV, and ADAV) and six days (for SFNV and SFSV) for the emergence of cytopathic effects. Neutralizing antibody titers were expressed at 20, 40, 80, and 160 serum dilutions, and titers ≥ 40 were interpreted as positive for stringency. 

We performed additional MN assays for Punique (PUNV) and Massilia (MASV) viruses (sandfly fever Naples serocomplex) in samples with detectable SFNV neutralizing antibodies. In these samples, two-fold serial dilutions from 1:20 to 1:1280 were tested as explained above. Samples with indeterminate MN results were omitted from the final datasets following repeat testing. All virus isolates were obtained from the European Virus Archive (https://www.european-virus-archive.com).

### 2.4. Statistical Analysis

Statistical analysis was conducted using the IBM SPSS 23.0 statistical package program. Data were summarized as frequencies and percentages. Relationships between categorical variables were determined via the Chi square test. The significance level was set at *p* < 0.05.

## 3. Results

We selected and tested 124 sera with 11 seroreactivity profiles for various phleboviruses in IFA (Table 1). Phlebovirus NAs were detected in a total of 50 samples (50/124, 40.3%), targeting TOSV, SFNV, and SFSV. Individual virus NAs were documented in 38 samples (38/50, 76%), while dual virus exposure in various combinations was observed in 12 (24%), with a prevalence of 9.6% (12/124). The break-up of the MN and the associated IFA profiles are provided in Table 2. Individual IFA and MN findings are provided in Supplemental Table S1. 

We compared virus-based NA prevalence and selected demographics and the predonation survey responses of the subjects. No statistically significant correlation was observed between NA detection of any virus and sex, age strata, location of residence, or history of frequent outdoor activity (Table 3). 

### 3.1. TOSV NA Seroprevalence Findings

TOSV NAs were present in a total of 28 samples (28/124, 22.6%) observed as the most frequently documented NAs in the study cohort (Table 1). TOSV-specific antibodies were the only detectable NAs in 19 sera (17/26, 65.3%), whereas SFNV or SFSV NAs were also present in the remaining samples (Table 2). They were further identified as the most prevalent single virus exposure in the study cohort (17/124, 13.7%). In sera with detectable NAs, antibody titers of 1/40, 1/80 and 1/160 were observed in 12 (42.8%), 4 (14.3%) and 12 (42.8%) samples, respectively (Table 4).

In comparisons with NA detection and IFA reactivity, TOSV NAs were significantly associated with TOSV IFA positivity, which also correlated with NA titers (Supplemental Tables S2 and S3 and Table 4). Similarly, reactivity in SFNV IFA showed a statistically significant difference in samples with low- and high-titer TOSV NAs, where high titers were correlated with reactivity, indicating probable cross-reactions. Interestingly, an association of TOSV NAs and SFSV IFA was further noted, where low NA titers were associated with SFSV IFA reactivity (Table 4). These findings indirectly suggest co-circulation of an SFSV-related virus with shared vectors with TOSV. 

### 3.2. SFNV NA Seroprevalence Findings

SFNV NAs were detected in 19 samples (15.3%) (Table 1) and in 9 (47.3%) as the single phlebovirus exposure (Table 2). In sera with detectable NAs, antibody titers of 1/40, 1/80, and 1/160 were observed in six (31.5%), five (26.3%), and eight (42.1%) samples, respectively (Table 4). A correlation with NA detection and SFNV IFA reactivity was observed, with a statistically significant increase in IFA positivity with elevated NA titers. No association of TOSV or other phlebovirus IFAs with SFNV NAs was noted (Supplemental Table S2 and S3, and Table 4). Additional MN tests using MASV and PUNV were negative.

### 3.3. SFSV NA Seroprevalence Findings

SFSV NAs were present in 15 samples (15/124, 12.1%) (Table 1) and in 8 (53.3%) as the single phlebovirus exposure. Distribution of NA titers noted as 1/40, 1/80, and 1/160 were found in six (40%), three (20%), and six (40%) samples, respectively. Similar to the observations for TOSV and SFNV, SFSV NA detection and IFA reactivity were correlated. A similar association was noted for SFCV IFA as well. A statistically significant increase in IFA positivity between low and high NA titers was further noted in SFSV but not in SFCV. An association of TOSV IFA and SFSV NA detection was also observed, but NA titer correlations could not be established due to the low number of reactive samples (Table 4). A similar trend was not present for SFNV IFA.

## 4. Discussion

In this study, we carried out an MN-based evaluation of sera from blood donors with previous evidence of phlebovirus exposure. For the screening and NA titer determination, we used established and several recently characterized viruses, a total of seven strains in MN assays, making this study the most comprehensive serological evaluation in a sandfly-borne phlebovirus endemic region.

We detected NAs to three distinct phleboviruses, namely TOSV, SFNV, and SFSV, in the study cohort. The most frequently observed virus exposure was TOSV, with a total prevalence of 20.9% and a single agent prevalence of 13.7%. Previous reports have already documented widespread TOSV exposure in Turkey, with neutralizing antibody prevalence of 2.9–14.4% in various cohorts, and comparable epidemiological features or risk factors reported for other endemic countries [12]. Despite the reporting of index cases in 2011 and the subsequent documentation of additional cases [13,14,15,16], TOSV can still be considered as a neglected agent of virally induced central nervous system infections in Turkey, as nucleic-acid- or serology-based diagnostics are not readily available for most healthcare establishments likely to see new cases. Furthermore, TOSV NAs and RNAs were reported in canine and feline sera, as well as tissues of various wild bird species, suggesting roles for nonhuman vertebrate and avian vectors in TOSV maintenance or dispersion in the region [19,20,21]. Hence, evidence from various sources including this study indicate TOSV circulation, which should be considered in infections with compatible symptoms.

We further observed SFNV and SFSV IFA reactivity to correlate with TOSV NA detection in the study. The SFNV findings are not surprising, as TOSV and SFNV are antigenically related, and serological cross-reactions are well documented [10,11]. However, SFSV findings require further elaboration. In the study, an association of TOSV IFA and SFSV NA, albeit a lack of correlation in NA titers, was also documented. Taken together, these findings suggest potential co-circulation of these viruses resulting in increased incidence of exposure, which may be due to the temporal or spatial activity of multiple virus-vectors in a given region as well as shared sandfly vectors. In general, the sandfly species *Phlebotomus perniciosus* and *Phlebotomus perfiliewi* sensu lato are considered efficient TOSV vectors, whereas SFSV is typically vectored by *Phlebotomus papatasi* [1,22]. However, the relationship between phleboviruses and vectors does not seem exclusive, and identical phleboviruses can be identified within several sandfly species [3]. Interestingly, in the only available vector screening effort with TOSV detection from Turkey, virus sequences were identified in *Phlebotomus papatasi*, *Phlebotomus major* complex, and *Sergentomyia dentata* species [23]. The sandflies belonging in the *Phlebotomus major* complex were also documented as the main vector for SFTV, the regional SFSV variant in Anatolia [24]. Therefore, our MN findings support the previous data from vector screening and suggest that TOSV and SFSV exposures coincide, possibly due to shared vectors or the presence of several sandfly species capable of transmitting both viruses. Data from several endemic regions further indicate that many species of sandfly could be competent for TOSV maintenance and transmission, likely to harbor and transmit multiple phleboviruses [22]. These findings should be corroborated in larger cohorts, where the shared vector hypothesis can be further supported by epidemiological data or virus detection in sandflies.

In the study, the prevalence of SFNV NAs was observed as 15.3%, with single exposure prevalence of 18%. Similar to the TOSV findings, SFNV NA detection and IFA reactivity were correlated but not to TOSV or other phlebovirus IFAs. Moreover, we could not demonstrate NAs of antigenically related ZERV, MASV, and PUNV in the study group or SFNV-NA-positive samples. Along with SFSV, SFNV is one of the historical agents of sandfly fever and has been endemic in the Mediterranean Basin, the Middle East, Central Asia, and Europe [25]. However, the last documented cases date back over three decades, and the virus has not been detected in any of the relatively recent field surveys around the Mediterranean Basin [3,25,26]. Similarly, SFNV exposure was reported from residents or blood donors from central, Mediterranean, and Aegean parts of Turkey, in specimens spanning from 1955 to 2009, via various methods, without acute or recent cases of SFNV infections or detection in sandflies [10,12]. Therefore, it is safe to assume that our detection of NAs represents previous exposure and SFNV may not be actively circulating in Turkey. The human exposure and potential public health impact of these newly described viruses still remain to be explored.

Finally, we detected exposure to the other well-established agent of sandfly fever—SFSV— in 12.1% of the samples, representing 8% as the single phlebovirus prevalence in the cohort. Here, SFSV and SFCV IFA reactivity were associated with SFSV NAs detection, and SFSV IFA reactivity significantly increased with NA titers, as observed for SFNV and TOSV. Similarly, an association of NA detection with TOSV IFA reactivity was observed; probable explanations with regards to sandfly vectors are provided above. In parallel with SFNV, historical and recent records of SFSV exposure are available from Turkey [10,12]. However, it was also identified as the causative agent in symptomatic cases via nucleic acid detection from various regions in Anatolia [27,28], indicating ongoing virus activity. SFSV and SFCV are closely-related to SFTV, the novel SFSV clade initially described in samples collected from individuals with febrile disease associated with sandfly exposure during 2007–2008 [17]. Subsequent reports have described potential vector sandflies and clinical symptoms, representing a considerably severe form of sandfly fever [18,24]. We could not perform parallel MN testing utilizing SFTV or SFCV due to the lack of available virus isolates, one of the main shortcomings of this study. Given the genome sequence similarities among isolates, some degree of cross-reactions should be expected to occur in serological assays targeting these viruses. However, it needs to be investigated whether cross-neutralizing antibodies detectable via MN are produced or persist in exposed individuals. In addition to sandfly fever, both SFSV and SFTV have been reported as causative agents in cases with symptoms involving the central nervous system, comparable to TOSV [29,30]. Hence, diagnostic and screening assays capable of detecting, and preferably discriminating, SFSV and related phleboviruses are needed.

We could not identify exposure to ADAV, ZERV, PUNV, or MASV in the study. ADAV was isolated from sandflies collected in Mediterranean Anatolia, with documented human exposure and a high prevalence of neutralizing antibodies in domestic animals in the region [31]. It has also been reported in dogs from Greece and Cyprus, indicating activity around the eastern Mediterranean [11]. Toros virus (TORV) and ZERV are additional viruses described in sandflies collected from locations in the vicinity of ADAV isolation and have not yet been tested for human exposure [32]. TORV is included in the SFSV serocomplex but remains distinct from the SFSV–SFTV–SFCV clade, being closely related to Corfou virus [32]. We could not test for TORV in this study due to limited sample availability for further testing. Our PUNV or MASV MN findings suggest the absence of these phleboviruses, which are in circulation in western parts of the Mediterranean [33,34]. Nevertheless, we identified NAs in various titers for multiple pathogenic phleboviruses of distinct serocomplexes in 9.6% of the samples, indicating individual exposure to several pathogenic phleboviruses, which was not reported from particular endemic regions with sufficient information [35]. Although timing and frequency of virus exposure are hard to assess using MN in a cross-sectional setting, the exposure patterns and antibody titers possibly suggest repeated TOSV and SFSV exposure in the study cohort [3,8]. Therefore, these viruses should be considered as potential etiological agents in individuals with compatible clinical presentations and in susceptible individuals as well as travel-related cases.

In conclusion, in an attempt to broadly characterize human exposure to several pathogenic phleboviruses from distinct serocomplexes, we identified TOSV, SFSV, and SFNV NAs as individual or combined markers of previous infections in a selected cohort of blood donors. No evidence of exposure to two local and two global viruses could be demonstrated. Diagnostic testing and serological screening efforts should be focused on these pathogens.

## Figures and Tables

**Table 1:** Immunofluorescence assays (IFA) profiles and microneutralization (MN) findings in the study cohort.

		Frequency	Prevalence
**IFA** (n: 124)	TOSV	33	26.6%
	SFSV	13	10.4%
	SFNV	5	4.0%
	SFCV	3	2.4%
	SFSV+SFCV	27	21.7%
	TOSV+SFNV	26	20.9%
	TOSV+SFNV+SFSV	2	1.6%
	TOSV+SFSV+SFCV	1	0.8%
	SFNV+SFSV+SFCV	3	2.4%
	TOSV+SFNV+SFCV	1	0.8%
	TOSV+SFNV+SFSV+SFCV	10	8%
**MN** (n: 124)	TOSV	28	22.6%
	SFNV	19	15.3%
	SFSV	15	12.1%
	ADAV	0	0.0%
	ZERV	0	0.0%
	PUNV (n: 11)	0	0.0%
	MASV (n: 11)	0	0.0%

**TOSV:** Toscana virus, **SFSV:** sandfly fever Sicilian virus, **SFNV:** sandfly fever Naples virus, **SFCV:** sandfly fever Cyprus virus, **ADAV:** Adana virus, **ZERV:** Zerdali virus, **PUNV:** Punique virus, **MASV:** Massilia virus.

**Table 2:** Distribution of Immunofluorescence assays (IFA) profiles in microneutralization (MN) reactive samples.

IFA Profile	MN Profile					
	TOSV (n: 19)	SFNV (n: 9)	SFSV (n: 10)	TOSV+SFNV (n: 7)	TOSV+SFSV (n: 2)	SFNV+SFSV (n: 3)
TOSV (n: 33)	6	0	0	1	0	0
SFNV (n: 5)	0	3	0	1	0	0
SFSV (n: 13)	0	0	4	0	0	2
SFCV (n: 3)	1	0	0	0	0	0
TOSV+SFNV (n: 26)	8	4	0	3	0	0
SFSV+SFCV (n: 27)	1	1	6	0	1	1
TOSV+SFNV+SFSV (n: 2)	1	0	0	0	0	0
SFNV+SFSV+SFCV (n: 3)	0	1	0	0	0	0
SFSV+ SFCV+TOSV+SFNV (n: 10)	2	0	0	2	1	0

**Table 3:** Association of microneutralization (MN) detection and demographics or possible risk factors of the study cohort.

		MN					
		TOSV-Positive (n: 28)	*p* Value	SFNV-Positive (n: 19)	*p* Value	SFSV-Positive (n: 15)	*p* Value
**Sex**	F (n: 4)	1	1.000	1	0.424	0	1.000
	M (n: 120)	27		18		15	
**Region**	Central Anatolia (n: 111)	28	0.038	19	0.121	15	0.364
	Black Sea (n: 13)	0		0		0	
Age strata *	20–29 (n: 44)	6	0.204	5	0.682	7	0.699
	30–39 (n: 33)	9		6		4	
	40–49 (n: 30)	7		4		3	
	50–59 (n: 17)	6		4		1	
**Frequent outdoor activity**	Yes (n: 75)	20	0.267	10	0.734	8	0.747
	No (n: 49)	8		9		7	

* Donor ages out of range (19 or ≥60) were added to the closest group (n: 7).

**Table 4:** Immunofluorescence assays (IFA) reactivity and neutralizing antibody (NA) titers according to phlebovirus strains.

			TOSV					SFNV					SFSV				
			Negative	Positive				Negative	Positive				Negative	Positive			
			0 and 1/20	1/40	1/80	1/160	*p* Value	0 and 1/20	1/40	1/80	1/160	*p* Value	0 and 1/20	1/40	1/80	1/160	*p* Value
**IFA**	TOSV	Positive	49	9	3	12	0.001	57	3	3	5	0.967	71	1	0	1	0.001
		Negative	45	3	1	0		38	3	2	3		38	5	3	5	
	SFNV	Positive	29	6	3	9	0.008	28	3	4	8	<0.001	45	1	0	1	0.113
		Negative	65	6	1	3		67	3	1	0		64	5	3	5	
	SFSV	Positive	46	5	2	1	0.036	44	4	2	1	0.158	41	6	3	6	<0.001
		Negative	48	7	2	11		51	2	3	7		68	0	0	0	
	SFCV	Positive	36	5	2	1	0.128	36	3	2	1	0.407	35	4	2	4	0.089
		Negative	58	7	2	11		59	3	3	7		74	2	1	2	

## Data Availability

All data presented in this study are available in the manuscript text or as supplements.

## Supplementary Materials

The following supporting information can be downloaded at: https://www.mdpi.com/article/10.3390/v15091902/s1, Table S1: Background information and IFA-MN data in individuals enrolled in the study; Table S2: Distribution of IFA and MN findings according to virus strains; Table S3: IFA intensities and NA titers according to virus strains.

## References

[1] Maroli M., Feliciangeli M.D., Bichaud L., Charrel R.N., Gradoni L. (2013). Phlebotomine Sandflies and the Spreading of Leishmaniases and Other Diseases of Public Health Concern. Med. Vet. Entomol..

[2] Kuhn J.H., Adkins S., Alkhovsky S.V., Avšič-Županc T., Ayllón M.A., Bahl J., Balkema-Buschmann A., Ballinger M.J., Bandte M., Beer M. (2022). 2022 Taxonomic Update of Phylum Negarnaviricota (Riboviria: Orthornavirae), Including the Large Orders Bunyavirales and Mononegavirales. Arch. Virol..

[3] Ayhan N., Charrel R.N. (2017). Of Phlebotomines (Sandflies) and Viruses: A Comprehensive Perspective on a Complex Situation. Curr. Opin. Insect Sci..

[4] Chaves L.F., Calzada J.E., Valderrama A., Saldaña A. (2014). Cutaneous Leishmaniasis and Sand Fly Fluctuations Are Associated with El Niño in Panamá. PLoS Negl. Trop. Dis..

[5] Lambert A.J., Hughes H.R. (2021). Clinically Important Phleboviruses and Their Detection in Human Samples. Viruses.

[6] Ergunay K. (2014). Phlebotomus Fever—Sandfly Fever. Emerging Infectious Diseases.

[7] Dionisio D., Esperti F., Vivarelli A., Valassina M. (2003). Epidemiological, Clinical and Laboratory Aspects of Sandfly Fever. Curr. Opin. Infect. Dis..

[8] Pierro A., Ficarelli S., Ayhan N., Morini S., Raumer L., Bartoletti M., Mastroianni A., Prati F., Schivazappa S., Cenni P. (2017). Characterization of Antibody Response in Neuroinvasive Infection Caused by Toscana Virus. Clin. Microbiol. Infect..

[9] Magurano F., Nicoletti L. (1999). Humoral Response in Toscana Virus Acute Neurologic Disease Investigated by Viral-Protein-Specific Immunoassays. Clin. Diagn. Lab. Immunol..

[10] Ergünay K., Litzba N., Lo M.M., Aydoǧan S., Saygan M.B., Us D., Weidmann M., Niedrig M. (2011). Performance of Various Commercial Assays for the Detection of Toscana Virus Antibodies. Vector Borne Zoonotic Dis..

[11] Alwassouf S., Christodoulou V., Bichaud L., Ntais P., Mazeris A., Antoniou M., Charrel R.N. (2016). Seroprevalence of Sandfly-Borne Phleboviruses Belonging to Three Serocomplexes (*Sandfly fever Naples*, *Sandfly fever Sicilian* and *Salehabad*) in Dogs from Greece and Cyprus Using Neutralization Test. PLoS Negl. Trop. Dis..

[12] Ergünay K., Polat C., Özkul A. (2020). Vector-Borne Viruses in Turkey: A Systematic Review and Bibliography. Antiviral Res..

[13] Ergünay K., Saygan M.B., Aydoǧan S., Lo M.M., Weidmann M., Dilcher M., Şener B., Hasçelik G., Pinar A., Us D. (2011). Sandfly Fever Virus Activity in Central/Northern Anatolia, Turkey: First Report of Toscana Virus Infections. Clin. Microbiol. Infect..

[14] Kuşcu F., Menemenlioğlu D., Oztürk D.B., Korukluoğlu G., Uyar Y. (2014). Acute Toscana Virus Infection in an Anti-HIV Positive Patient. Mikrobiyol. Bul..

[15] Erdem H., Ergunay K., Yilmaz A., Naz H., Akata F., Inan A.S., Ulcay A., Gunay F., Ozkul A., Alten B. (2014). Emergence and Co-Infections of West Nile Virus and Toscana Virus in Eastern Thrace, Turkey. Clin. Microbiol. Infect..

[16] Okar S.V., Bekircan-Kurt C.E., Hacıoğlu S., Erdem-Özdamar S., Özkul A., Ergünay K. (2020). Toscana Virus Associated with Guillain–Barré Syndrome: A Case–Control Study. Acta Neurol. Belg..

[17] Çarhan A., Uyar Y., Özkaya E., Ertek M., Dobler G., Dilcher M., Wang Y., Spiegel M., Hufert F., Weidmann M. (2010). Characterization of a Sandfly Fever Sicilian Virus Isolated during a Sandfly Fever Epidemic in Turkey. J. Clin. Virol..

[18] Kocak Tufan Z., Weidmann M., Bulut C., Kinikli S., Hufert F.T., Dobler G., Demiroz A.P. (2011). Clinical and Laboratory Findings of a Sandfly Fever Turkey Virus Outbreak in Ankara. J. Infect..

[19] Dincer E., Gargari S., Ozkul A., Ergunay K. (2015). Potential Animal Reservoirs of Toscana Virus and Coinfections with Leishmania Infantum in Turkey. Am. J. Trop. Med. Hyg..

[20] Dincer E., Karapinar Z., Oktem M., Ozbaba M., Ozkul A., Ergunay K. (2016). Canine Infections and Partial S Segment Sequence Analysis of Toscana Virus in Turkey. Vector-Borne Zoonotic Dis..

[21] Hacioglu S., Dincer E., Isler C.T., Karapinar Z., Ataseven V.S., Ozkul A., Ergunay K. (2017). A Snapshot Avian Surveillance Reveals West Nile Virus and Evidence of Wild Birds Participating in Toscana Virus Circulation. Vector-Borne Zoonotic Dis..

[22] Ayhan N., Prudhomme J., Laroche L., Bañuls A.L., Charrel R.N. (2020). Broader Geographical Distribution of Toscana Virus in the Mediterranean Region Suggests the Existence of Larger Varieties of Sand Fly Vectors. Microorganisms.

[23] Özbel Y., Oğuz G., Arserim S.K., Erişöz Kasap Ö., Karaoglu B., Yilmaz A., Emanet N., Günay F., Hacioğlu S., Demirok M.C. (2020). The Initial Detection of Toscana Virus in Phlebotomine Sandflies from Turkey. Med. Vet. Entomol..

[24] Ergunay K., Erisoz Kasap O., Kocak Tufan Z., Turan M.H., Ozkul A., Alten B. (2012). Molecular Evidence Indicates That *Phlebotomus major sensu lato* (Diptera: Psychodidae) Is the Vector Species of the Recently-Identified Sandfly Fever Sicilian Virus Variant: Sandfly Fever Turkey Virus. Vector-Borne Zoonotic Dis..

[25] Alkan C., Bichaud L., De Lamballerie X., Alten B., Gould E.A., Charrel R.N. (2013). Sandfly-Borne Phleboviruses of Eurasia and Africa: Epidemiology, Genetic Diversity, Geographic Range, Control Measures. Antivir. Res..

[26] Eitrem R., Vene S., Niklasson B. (1990). Incidence of Sand Fly Fever among Swedish United Nations Soldiers on Cyprus during 1985. Am. J. Trop. Med. Hyg..

[27] Guler S., Guler E., Caglayik D.Y., Kokoglu O.F., Ucmak H., Bayrakdar F., Uyar Y. (2012). A Sandfly Fever Virus Outbreak in the East Mediterranean Region of Turkey. Int. J. Infect. Dis..

[28] Temocin F., Sari T., Tulek N. (2016). Sandfly Fever with Skin Lesions: A Case Series from Turkey. J. Arthropod. Borne. Dis..

[29] Becker M., Zielen S., Schwarz T.F., Linde R., Hofmann D. (1997). Pappataci-Fieber. Klin. Padiatr..

[30] Ergunay K., Ismayilova V., Colpak I.A., Kansu T., Us D. (2012). A Case of Central Nervous System Infection Due to a Novel Sandfly Fever Virus (SFV) Variant: Sandfly Fever Turkey Virus (SFTV). J. Clin. Virol..

[31] Alkan C., Alwassouf S., Piorkowski G., Bichaud L., Tezcan S., Dincer E., Ergunay K., Ozbel Y., Alten B., de Lamballerie X. (2015). Isolation, Genetic Characterization, and Seroprevalence of Adana Virus, a Novel Phlebovirus Belonging to the Salehabad Virus Complex, in Turkey. J. Virol..

[32] Alkan C., Erisoz Kasap O., Alten B., de Lamballerie X., Charrel R.N. (2016). Sandfly-Borne Phlebovirus Isolations from Turkey: New Insight into the *Sandfly fever Sicilian* and *Sandfly fever Naples* Species. PLoS Negl. Trop. Dis..

[33] Charrel R.N., Moureau G., Temmam S., Izri A., Marty P., Parola P., Da Rosa A.T., Tesh R.B., De Lamballerie X. (2009). Massilia Virus, a Novel Phlebovirus (Bunyaviridae) Isolated from Sandflies in the Mediterranean. Vector Borne Zoonotic Dis..

[34] Sakhria S., Bichaud L., Mensi M., Salez N., Dachraoui K., Thirion L., Cherni S., Chelbi I., De Lamballerie X., Zhioua E. (2013). Co-Circulation of Toscana Virus and Punique Virus in Northern Tunisia: A Microneutralisation-Based Seroprevalence Study. PLoS Negl. Trop. Dis..

[35] Percivalle E., Cassaniti I., Calzolari M., Lelli D., Baldanti F. (2021). Thirteen Years of Phleboviruses Circulation in Lombardy, a Northern Italy Region. Viruses.

